# Robot-assisted laparoscopic surgery after placing a self-expanding metallic stent for malignant rectal obstruction: a case report

**DOI:** 10.1186/s40792-019-0719-1

**Published:** 2019-10-25

**Authors:** Hiroshi Takeyama, Katsuki Danno, Takahiko Nishigaki, Masafumi Yamashita, Masami Yamazaki, Tsuyoshi Yamakita, Akihiro Nishihara, Hirokazu Taniguchi, Masayo Mizutani, Itsuko Nakamichi, Mamoru Yura, Kimimasa Ikeda, Yoshio Oka

**Affiliations:** 1 Department of Gastroenterological Surgery, Minoh City Hospital, Kayano 5-7-1, Minoh, Osaka 562-0014 Japan; 2 Department of Gastroenterology, Minoh City Hospital, Osaka, 562-0014 Japan; 3 Department of Pathology, Minoh City Hospital, Osaka, 562-0014 Japan

**Keywords:** Robot-assisted laparoscopic surgery, Rectal cancer, Self-expanding metallic stent, Self-expandable metallic stent, Obstruction, Malignant rectal obstruction, Malignant colorectal obstruction

## Abstract

**Background:**

Approximately 20% of colorectal cancer patients show complete or incomplete bowel obstruction as an early symptom. Preoperative nonsurgical decompression such as placing a self-expanding metallic stent for malignant colorectal obstruction has been shown to be effective for reducing perioperative morbidity and mortality. However, there is a lack of published studies reporting robot-assisted laparoscopic surgery (RALS) after self-expanding metallic stent (SEMS) placement for malignant rectal obstruction (MRO). To our knowledge, this is the first report to do so.

**Case presentation:**

An 80-year-old man with incomplete paralysis of the lower limbs as well as bladder–rectal disorder due to a spine fracture sustained in a fall accident 26 years ago presented with lower abdominal pain and vomiting. Abdominal multi-detector computed tomography revealed an obstructive rectal tumor with distended bowel on the oral side. Emergency colonoscopy was performed, and an SEMS placed. The patency of SEMS and decompression of the distended bowel was confirmed, and elective RALS was performed 29 days after SEMS placement. To our knowledge, this is the first report of RALS after decompression with SEMS placement for MRO.

**Conclusions:**

RALS after SEMS placement is a safe and feasible therapeutic strategy for MRO.

## Background

For malignant colorectal obstruction (MCO), preoperative decompression by placing a self-expanding metallic stent (SEMS) has been recognized as a safe and effective therapeutic strategy, and there is ample evidence for the feasibility of laparoscopic surgery after SEMS placement [1, 2]. However, no studies have reported robot-assisted laparoscopic surgery (RALS) after SEMS placement for malignant rectal obstruction (MRO).

We report a case of RALS after SEMS placement for MRO. To the best of our knowledge, no similar cases have been reported in the literature and this is the first report of RALS after SEMS placement for MRO.

## Case presentation

**Fig. 1:**
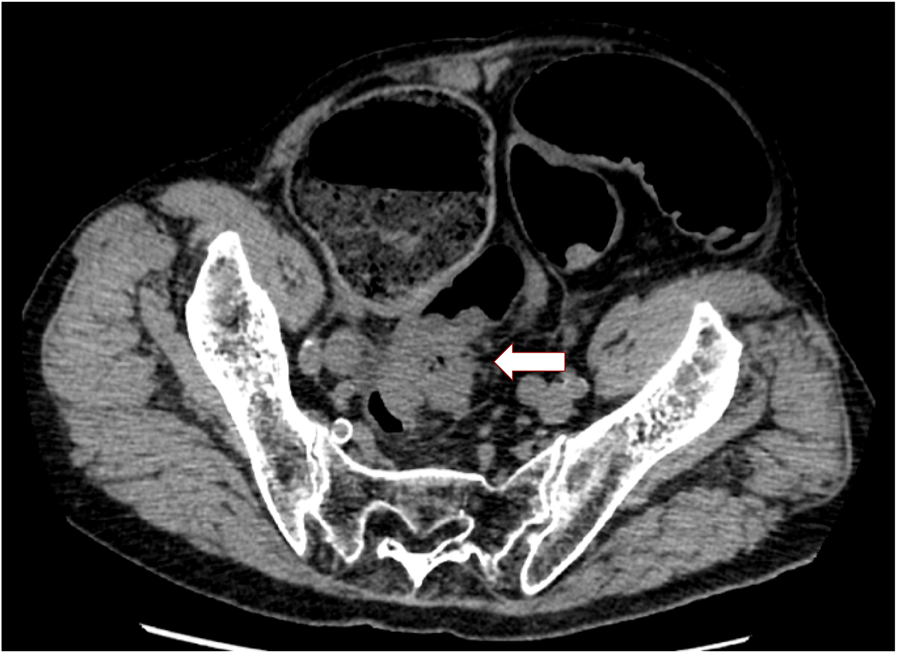
Abdominal multi-detector computed tomography scan. Rectal tumor (white arrow) and distended bowel on the oral side can be seen

**Fig. 2:**
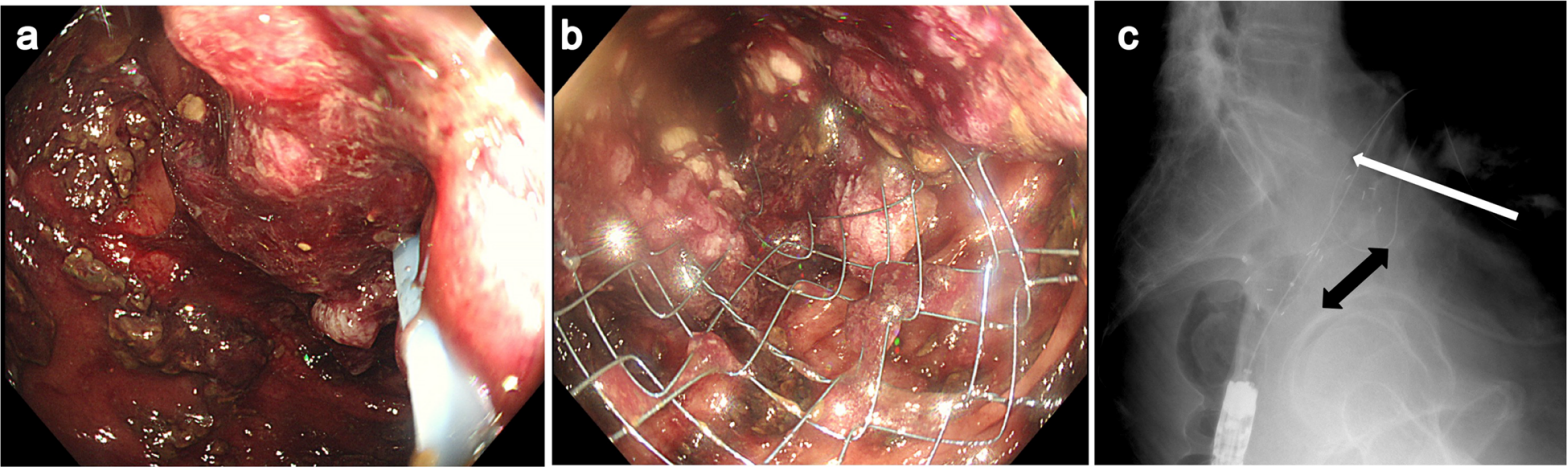
**a** Colonoscopy showing bowel obstruction with a malignant rectal tumor. **b** Self-expanding metallic stent placement through the malignant rectal obstruction using endoscopy. **c** Fluoroscopy showing a 2.8-cm constricted area due to tumor (black double-headed arrow) which of the upper end located at the anal side of the promontory (white long arrow)

**Fig. 3:**
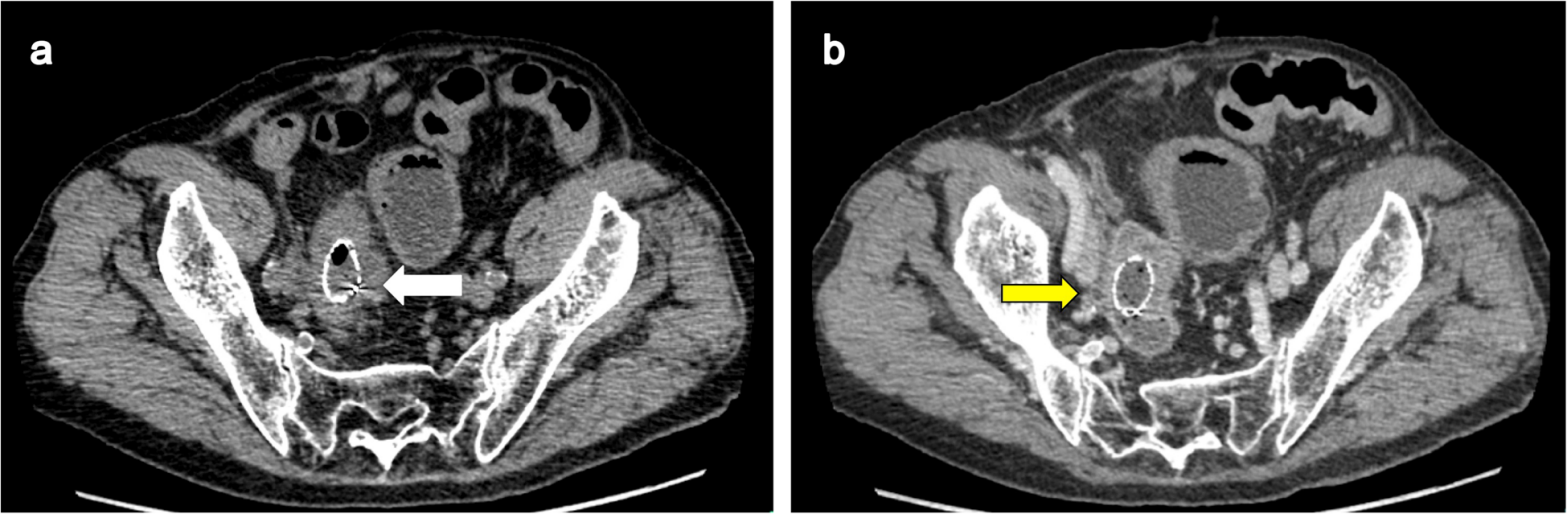
**a** Abdominal multi-detector computed tomography (CT) scan showing decompression of distended bowel and patency of self-expanding metallic stent (white arrow). **b** A contrast-enhanced CT image showing the existence of a tumor near the right ureter, with possible invasion (yellow arrow)

An 80-year-old man who presented with lower abdominal pain and vomiting and was admitted to the Minoh City Hospital (Osaka, Japan) in February 2019 exhibited incomplete paralysis of the lower limbs and bladder–rectal disorder due to a spine fracture sustained in a fall accident 26 years ago. Abdominal multi-detector computed tomography (CT) revealed a rectal tumor and obstruction with bowel distension on the oral side (Fig. 1). Emergency colonoscopy revealed obstruction due to a rectal malignant tumor; a biopsy specimen of the tumor was collected. Simultaneously, to decompress the distended bowel, an SEMS was emergently placed under endoscopy and fluoroscopy (Fig. 2a–c) using an 18 × 60-mm HANAROSTENT® Naturfit™ (Boston Scientific, Natick, MA, USA) stent. Endoscopy and fluoroscopy revealed that the lower end of the malignant tumor was located at a distance of 13 cm from the anal verge, whereas the upper end of the tumor was located at the anal side of the promontory. Thus, the location of the tumor was found to be the upper rectum (Fig. 2c). Histopathological examination showed the biopsy specimen to be a moderately differentiated tubular adenocarcinoma. After SEMS placement, decompression of the distended bowel and patency of SEMS was observed on a CT image, allowing for the oral intake of drugs, fluids, and diet (Fig. 3a). A contrast-enhanced CT image revealed a tumor near the right ureter with possible invasion (Fig. 3b), negative lymph node, and negative distant organ metastasis. Preoperative stage classification according to the Union for International Cancer Control (UICC) stage classification was T4a, N0, M0, stage IIB [3].

**Fig. 4:**
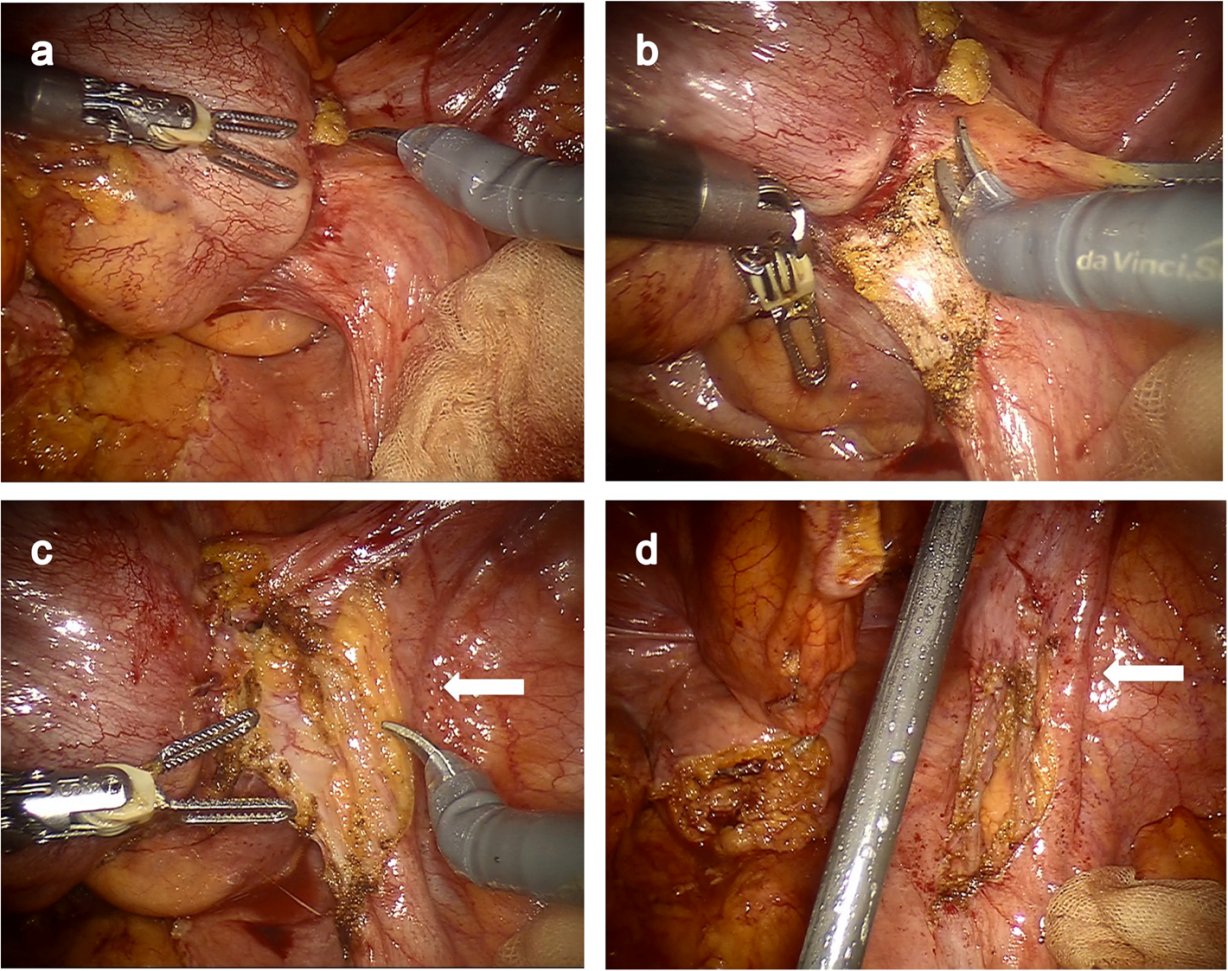
Intraoperative findings. **a** Laparoscopic view showing tumor fixation to right pelvic wall. **b**, **c** Articulated flexible robotic instruments helped create a stable surgical view, and the tumor could be approached from ideal directions, securing definitive negative margin avoiding injury to the right ureter (white arrow). **d** After the resection of the tumor from right side pelvic wall without injury to the right ureter (white arrow)

The patients who had incomplete paralysis of the lower limbs and bladder–rectal disorder selected Hartman’s procedure without reconstruction. Furthermore, we selected Hartman’s procedure considering the patient’s condition, his performance status, and age. Elective curative Hartman’s procedure was planned using the surgical robot system (da Vinci Si Surgical System; Intuitive Surgical, Inc., Sunnyvale, CA, USA). The patient was discharged from the hospital 14 days after SEMS placement, followed by elective admission for surgery 24 days after SEMS placement. A double-J catheter was placed in the right ureter to avoid intraoperative injury, and elective RALS was performed 29 days after SEMS placement (Fig. 4a–d).

Colonic and pelvic phase procedures were performed using the da Vinci surgical robot system with a single docking technique from the left caudal direction of the patient. Inferior mesenteric artery was ligated, and D3 lymph node dissection was performed. The operation time was 402 min, including 147 min of console time, and the estimated blood loss was 60 ml. Due to intestinal adhesion, it took 149 min from the start of operation to the start of console operation.

**Fig. 5:**
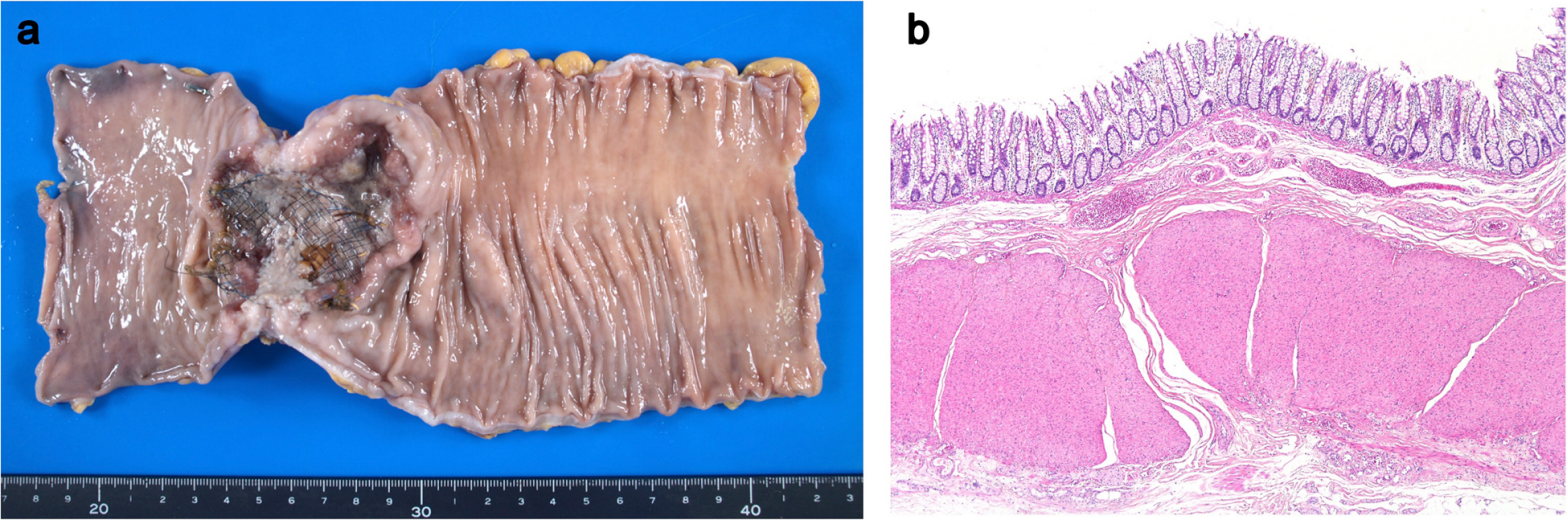
**a** Image of the surgical specimen showing a circumferential rectal tumor with placement of self-expanding metallic stent. **b** Histopathological image showing the absence of intestinal edema on the oral side (× 40, H&E stain)

Histopathologically, the tumor was diagnosed as T4a, N0 (0/12), M0, stage IIB, according to UICC classification [3]. In addition, histopathological examination showed that the tumor was resected with negative resection margins (R0-resection) and revealed the absence of intestinal edema, on the basis of edema criteria, on the oral side [1] (Fig. 5a, b). The patient received postoperative rehabilitation and was discharged 20 days after RALS with no postoperative complications.

## Conclusions

Complete or incomplete bowel obstruction presents as the first symptom in approximately 20% of colorectal cancer patients, and Lee et al. have reported that the frequency of MRO is approximately one third of that of malignant left colonic obstruction [4]. Traditionally, emergency surgery without preoperative decompression, accompanied by stoma formation, has been the most common treatment strategy for MCO [5]. Recently, preoperative nonsurgical decompression such as placing an SEMS for MCO has been increasingly performed, with accumulated studies reporting that SEMS placement is effective for decompression and can be a bridge to elective surgery; this approach has reduced perioperative morbidity and mortality with a significantly increased success rate for one-stage anastomosis [1, 6].

Use of laparoscopic surgery and one-stage anastomosis for MCO has been controversial because of the high morbidity due to edematous, fragile bowel and insufficient intraabdominal working space as a result of the distended bowel [7]. However, recent studies have concluded that preoperative decompression using SEMS and transanal drainage tube is helpful when performing subsequent elective laparoscopic surgery and one-stage anastomosis [8]. Preoperative decompression of the distended bowel increases intraabdominal working space during surgery and reduces bowel edema, leading to greater safety during one-stage anastomosis [1, 9].

On the basis of this knowledge, we thought that RALS as well as conventional laparoscopic surgery can be safely performed after the decompression for MRO. In Japan, since April 2018, the procedure has been covered under insurance, resulting in an increased and widespread use of RALS for rectal cancer treatment. In general, RALS has an edge over conventional laparoscopic surgery due to the former’s ability to allow delicate operations with a stable operating view of the deep and narrow pelvis with the three dimensional (3D)-view scope and the flexible instruments on the robot. We expected these advantages to lead to a safer rectal surgery, and therefore, we selected RALS over conventional laparoscopic surgery.

Although the feasibility of laparoscopic resection of pT4 rectal cancer has recently been reported, enough evidence for the using of conventional laparoscopic surgery for advanced-stage rectal cancer is still lacking [10]. Touching the tumor with laparoscopic instruments during surgery should be strictly avoided based on the “no touch” principle; this is one of the reasons why the use of laparoscopy for advanced-stage colorectal cancer remains controversial [11]. Articulated flexible robotic instruments could impart safety during surgery by avoiding contact with the tumor. Indeed, Crolla et al. have recently reported the feasibility of robot-assisted laparoscopic resection of clinical T4b tumors of the distal sigmoid and rectum [12].

In robot-assisted partial nephrectomy (RAPN) for renal tumors, articulated flexible instruments allow approach to the tumors from all directions with extremely useful visibility offered by 3D-vision scope so as to secure definitive surgical margin from the tumor, thereby reducing positive surgical margin [13]. In our patient with advanced MRO, tumor invasion into the right ureter was suspected on the basis of preoperative CT findings, and RALS provided a definitive negative surgical margin avoiding any injury to the ureter; thus, we could benefit from the advantages conferred by the robot-assisted surgery, similar to RAPN over conventional laparoscopic surgery. In fact, the malignant rectal tumor was resected without excess or insufficient margins, preventing any injury to the right ureter.

In our patient, who had bladder–rectal disorder, Hartman’s procedure, without reconstruction, was selected not only due to the patient’s condition but also because of his performance status and age. Because of these reasons, we did not perform one-stage anastomosis without covering stoma formation, although the resected specimen showed improved intestinal edema and its feasibility is expected in RALS as well as in conventional laparoscopic surgery for MRO after SEMS placement [1, 8].

There are some limitations of the study. First, the insertion of SEMS for MRO has several drawbacks. One of which is the location of implanted SEMS. Fortunately, the present case involved the upper rectum; the insertion of SEMS in the middle and lower rectum is still controversial. To date, SEMS placement is considered unsuitable for MRO near the anal verge (i.e., within 5 cm), given the potential of anal pain, foreign body sensation, tenesmus, and incontinence because of rectal irritation by the stent [14, 15]. Recently, Lee et al. reported the efficacy and safety of SEMS placement for MRO, including six bridges in the surgical cases of lower rectal obstruction 5 cm or less distant from the anal verge [4]. However, to obtain an appropriate distal margin of 2 cm or more during curative operation for lower rectal cancer, SEMS placement for MRO near the anal verge (i.e., within 5 cm) must be carefully considered. Second, one of drawbacks of robotic surgery is the absence of tactile sensation. Operators acquire only visual information and should proceed with the operation based on the visual information. Although we think that the visual information complements the absence of tactile sensation to a certain extent, the operator should pay greater attention to the exposure of the surgical field with SEMS-inserted organ during RALS than that during a conventional laparoscopic surgery for advanced rectal cancer, strictly based on the “no touch” principle. These points must be considered by surgeons when deciding an appropriate treatment.

Our results show that RALS after SEMS placement is a safe and feasible therapeutic strategy for MRO, allowing sufficient preoperative decompression using SEMS. This approach creates working space during RALS and improves intestinal edema. Further studies are expected to establish the superiority and feasibility of RALS after SEMS placement for MRO over other approaches.

## Data Availability

The datasets supporting the conclusions of this article are included within the article.

## Abbreviations

CT: Computed tomography
MCO: Malignant colorectal obstruction
MRO: Malignant rectal obstruction
RALS: Robot-assisted laparoscopic surgery
RAPN: Robot-assisted partial nephrectomy
SEMS: Self-expanding metallic stent
UICC: Union for International Cancer Control
